# Evolution and morphology of a contourite depositional system based on new evidence from 3D-seismic data offshore Madagascar (Paleogene, Morondava Basin)

**DOI:** 10.1038/s41598-022-24573-z

**Published:** 2022-11-19

**Authors:** F. Javier Hernández-Molina, Gabor Tari, Nicola Scarselli, Hoby Raharisolofo, Sophie F. Rahajarivelo, Adam Kirby, Wouter de Weger, Estefania Llave, Adrien Mergnat

**Affiliations:** 1 Department of Earth Sciences, Royal Holloway University of London, Egham, TW20 0EX Surrey UK; 2 OMV Exploration and Production GmbH, Trabrennstrasse 6-8, 1020 Vienna, Austria; 3 OMNIS, Lalana Razanakombana 21, 101 Antananarivo, Madagascar; 4 Instituto Geológico y Minero de España (IGME, CSIC), Rios Rosas 23, 28003 Madrid, Spain

**Keywords:** Ocean sciences, Sedimentology

## Abstract

Numerous bottom current-controlled depositional and erosional features, which together form Contourite Depositional Systems (CDS), have been recognized in deep-water settings over the past decade. Most of these systems are described based on two-dimensional (2D) seismic data, whereas only a few CDS have been characterised from high-resolution 3D data. Here we document a newly identified CDS that formed during the Paleocene within the Morondava Basin, offshore west Madagascar, through analysis of a depth-migrated 3D seismic survey, enhanced by the implementation of seismic attributes. Three seismic units (SU) mark the main evolutionary stages of the CDS: (a) the onset (SU1), (b) drift growth (SU2), and (c) burial (SU3) stages. The growth stage documents lateral upslope migration of a mounded drift and its associated moat. The increasing, long-term influence of bottom currents along the foot of the slope occurred simultaneously with plate tectonic, climatic and oceanographic changes. Evidence amassed from the CDS highly erosive bounding discontinuities, internal discontinuities, and moat architecture all indicate the intermittent behaviour of the currents over shorter time frames during its formation. Drift deposits form under the influence of weaker currents, while discontinuities appear to record the most vigorous currents, producing the large-scale morphology of the system.

## Introduction

Pioneer research that sought to define contourite drifts linked such features to the depositional action of bottom currents^1^. Recent decades have witnessed a proliferation of information on contourite drifts both in modern settings and in the ancient sedimentary record, wherein drifts and contourites synonymously refer to accumulations of sediment deposited by or significantly affected by bottom currents^2^. Altogether, researchers have proposed different types of drifts^2,3^, all having formed in areas with low (< 20 cm s^–1^) bottom current velocities^4^.

Besides drifts, erosive features such as contourite channel-like features commonly form in areas affected by enhanced bottom currents with higher velocities^5^. Depositional and erosional features frequently occur together, forming a Contourite Depositional System (CDS)^6^. These large features readily appear in 2D seismic reflection profiles and bathymetric data owing to their distinctive morphologies, seismic characteristics and overall architecture relative to the basin scale. Examples of contourites from 3D seismic datasets, however, are limited^7–9^. Information that can only be obtained from 3D seismic datasets—such as internal sedimentary stacking patterns, 3D morphologies and dimensions, types of deposits and internal architectures of the contourite channels—therefore remains scarce. Rigorous three-dimensional characterisation of these features may provide critical information for an integral picture of ancient bottom currents, providing new insights into past ocean circulation and climate. Furthermore, their potential coarse-grained infill renders them as reservoir targets for hydrocarbon exploration and subsurface storage.

**Figure 1:**
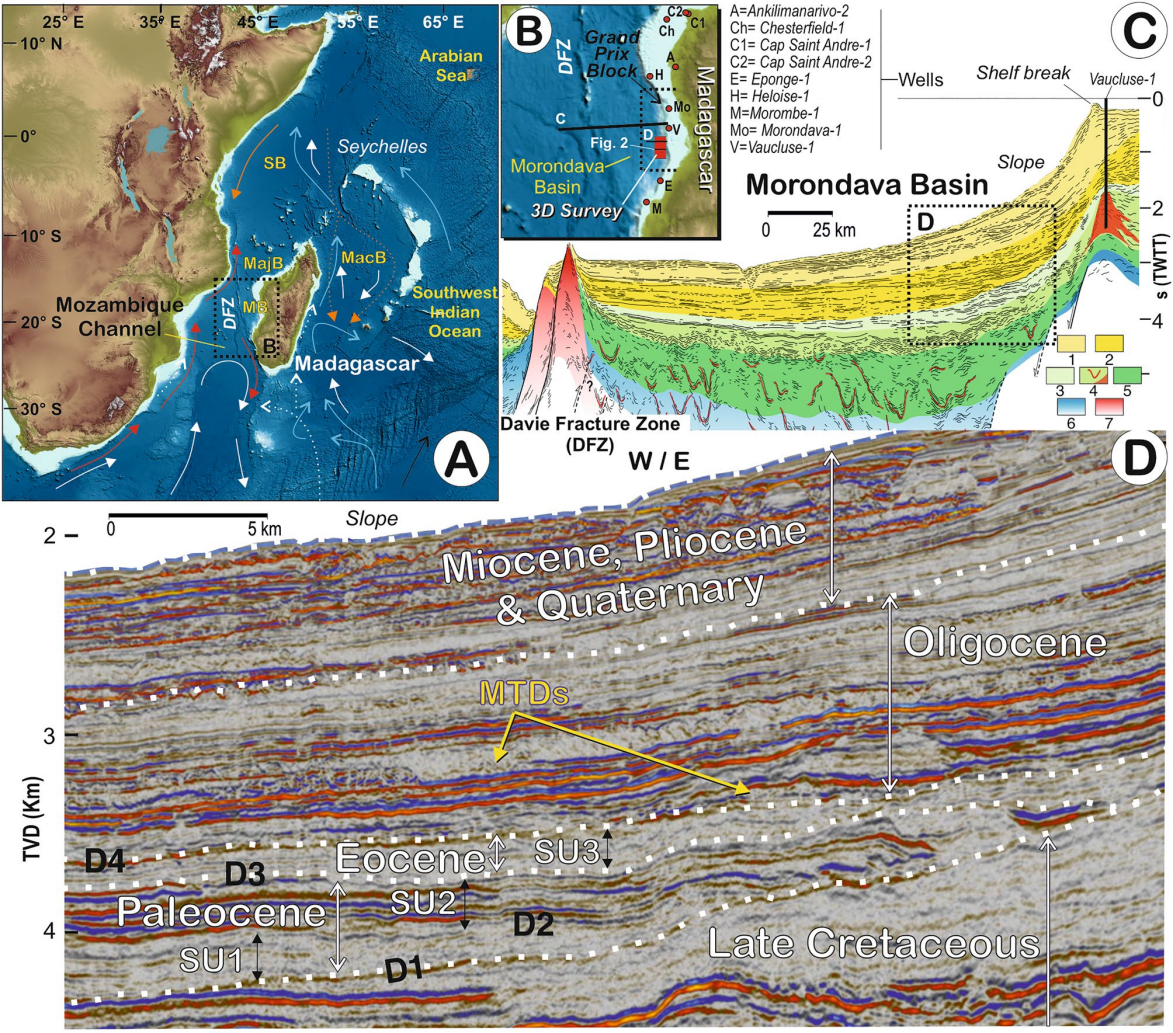
Location of the Morondava Basin with the present-day water mases circulation (**A**), dataset analysed from the Grand Prix bloc (**B**), regional setting (**C**) and chronostratigraphy of the Paleocene deposits studied (**D**). 1 = upper Cenozoic; 2 = lower Cenozoic; 3 = Senonian; 4 = Ceno-Turonian with sills and volcanics; 5 = lower Cretaceous; 6 = Jurassic (Upper Karoo); and 7 = Basement. General and simplified sketch showing circulation of the intermediate and deep water-masses^18^. The deep-water masses of the Mozambique Channel consist of the North Atlantic Deep Water (NADW) and the Antarctic Bottom Water (AABW), which is deflected to form a southerly flowing current (**A**). The NADW is partially blocked from spreading north by the Davie Fracture Zone (DFZ). Legend for the sedimentary basins: *MB* Morondava Basin, *MacB* Macarene Basin, *MajB*  Majunga Basin, *SB* Somali Basin. Legend for the regional deep-water masses*:* red arrows = North Atlantic deep water (NADW); light blue arrows = circumpolar deep water (CDW); orange arrows =  Red Sea water (RSW); orange dot arrows = Indian deep water (IDW); white dot arrows = Antarctic intermediate water (AAIW); and white arrows = Antarctic bottom water (AABW). Map from (**A**) based on the GEBCO_2022 data set (https://www.gebco.net/data_and_products/gridded_bathymetry_data/).

This study investigates the Paleogene sedimentary succession of the Morondava Basin offshore Madagascar (Mozambique Channel), within a recently acquired, high-quality, depth-migrated 3D seismic dataset (the Grand Prix block) that covers 3014 km^2^ (Fig. 1). The objective of this study is to characterise a new CDS in the Morondava Basin, comparing it to others described in the literature, so as to determine factors controlling a contourite system’s morphology. Conceptual and economic implications are also discussed.

## Regional setting

The Morondava Basin is located within the Mozambique Channel, along the western coast of Madagascar (Fig. 1). The basin is bounded to the west by the ridge of the Davie Fracture Zone (DFZ), an inactive Mesozoic transform fault; and to the north by a series of submarine volcanoes that form the border with the Majunga Basin (Fig. 1). Previous knowledge of the Morondava Basin is based on outcrop studies, seismic surveys and borehole information^10^. The basin formed during the Mesozoic break-up of Gondwana, coinciding with the inception of the Indian Ocean and the Mozambique Channel^11^. Among numerous tectonic events and associated features, the DFZ represents the main mechanism behind the motion of the Madagascar-India block^12^. The area developed in four phases^13,14^: (1) a *pre-rift* phase that occurred during the Carboniferous; (2) a *syn-rift* phase that started in the Permo-Triassic and persisted into the Jurassic, during which extensional grabens were likely infilled by lacustrine and continental deposits; (3) a *syn-rift-*drift phase that began in the middle Jurassic and continued into the Paleogene, bringing about the deposition of marine clastic units and carbonate deposition during periods of restricted-marine conditions; and finally, (4) a *passive-margin* phase that began in the late Paleogene and has continued until the present.

The stratigraphic section recorded in the Morondava Basin generally correlates to sections found in Mozambique and Tanzania^13,15^, although the Paleogene deposits in Madagascar are not as well documented due to the lack of deep-water wells. Paleocene deposits do not occur in the northern sector of the Morondava Basin, but can reach thicknesses of ~ 0.75 km in its southern and central sectors, where the succession is comprised of limestones, dolomites and marls^16,17^.

The deep-water masses of the Mozambique Channel consist at present day of the North Atlantic Deep Water (NADW) and the Antarctic Bottom Water (AABW)^18^, which is deflected to form a southerly flowing current (Fig. 1A). The NADW is partially blocked from spreading north by the Davie Fracture Zone (DFZ)^18^.

## Seismic analysis

**Figure 2:**
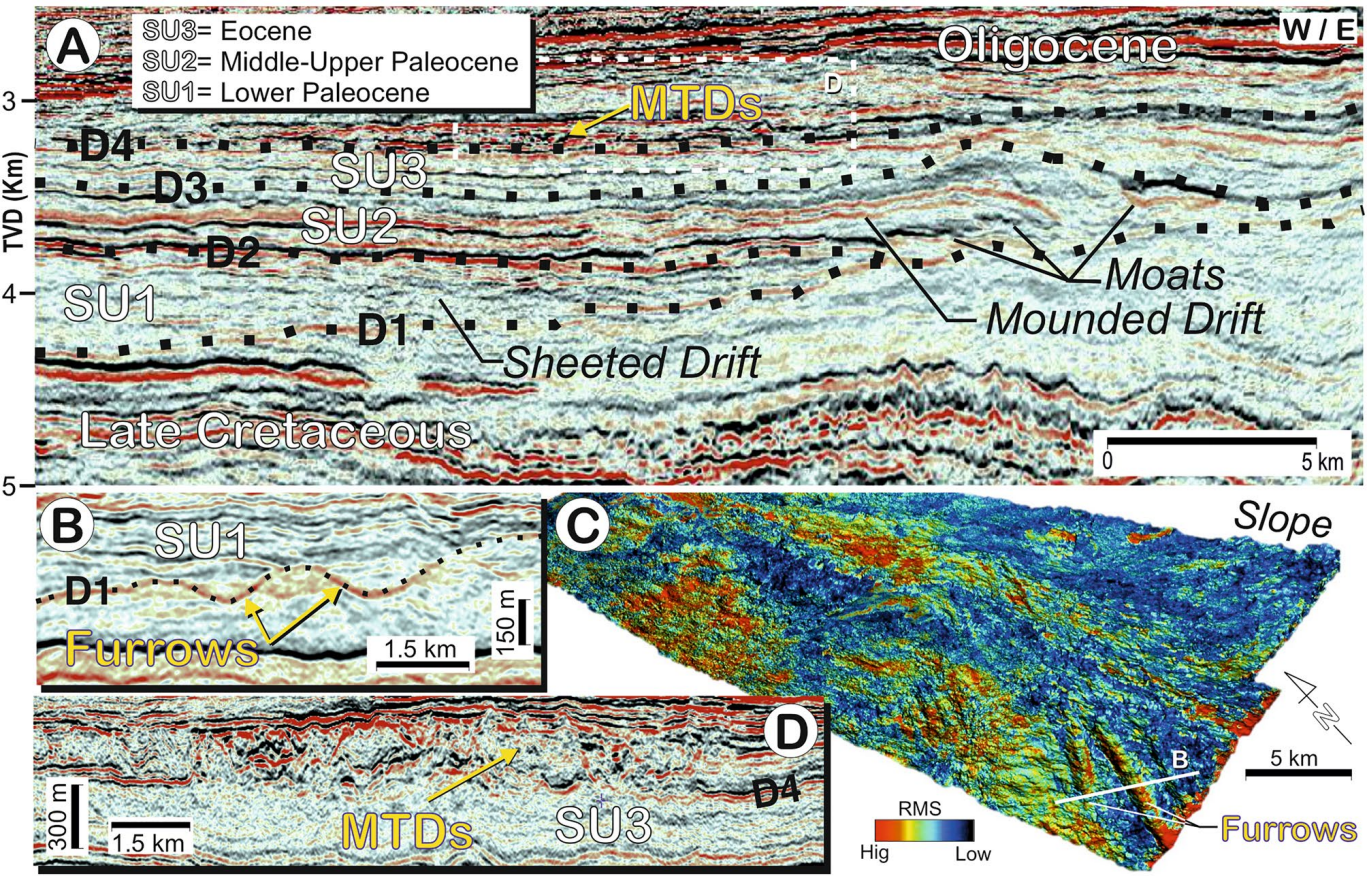
(**A**) 3D *seismic lines* indicating the seismic units, discontinuities and their chronology. (**B**) Furrows identified at basal discontinuity D1 with seismic lines, (**C**) the root mean square (RMS) amplitude attribute extraction for surface D1. (**D**) Example of the Mass Transport Deposits (MTDs) over discontinuity D4. Seismic line location in Fig. 1B.

The studied deposits in the Morondava Basin are characterised by major discontinuities (D1-D4) and present a predominantly layered internal acoustic reflection pattern, with significant upslope accretion (Figs. 1 and 2). Individual reflections display either onlapping or downlapping terminations upon the several discontinuities, while deposits feature an aggradational to an upslope progradational seismic reflection configuration.

The basal discontinuity (D1) marks a change in seismic facies and forms a major onlap surface. This discontinuity is a prominent regional reflection that shows evidence of erosion in seismic profiles throughout the area, having locally incised channel-like features trending SW, oblique to the 30°–35° trend of the main slope. These channelised features, initiated at the base of the slope, span under ~ 1 km in width, at least 5–10 km in length, and are incised to depths of ~ 0.1 km (Fig. 2). Above D1, three major regional seismic units (SU1 to SU3; Figs. 1 and 2) are observed, being bounded by the aforementioned discontinuities (D1–D4).

SU1, which directly overlies D1, has a tabular morphology and an average sedimentary thickness of 0.15 km; the unit appears as aggradational deposits outlined by low-amplitude reflections onlapping D1. The seismic facies are parallel, laterally continuous reflections, and uniformly distributed. In general, a weak response is observed at the base, increasing upward in the sequence, evolving towards high amplitude, laterally extensive reflections at the top (Fig. 2).

**Figure 3:**
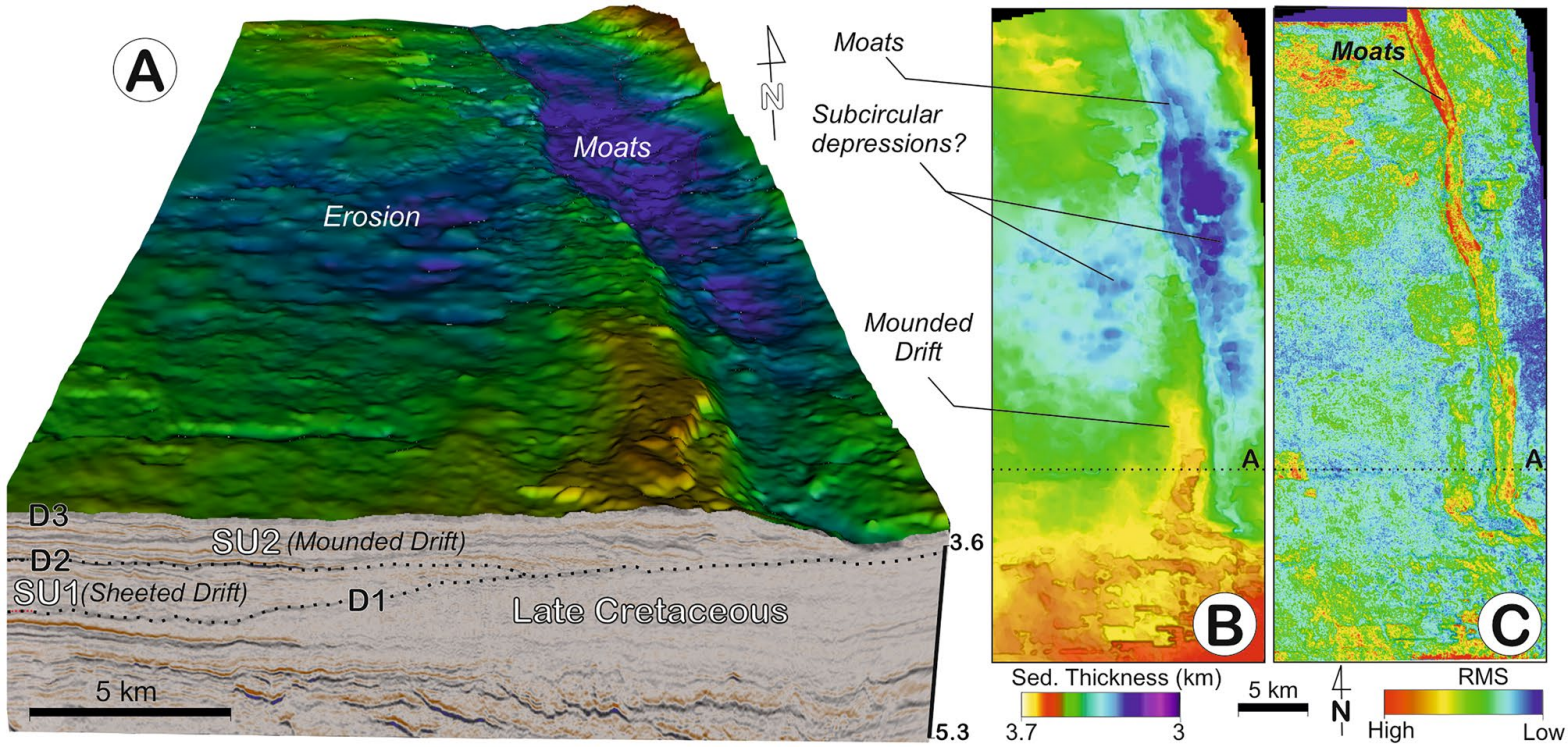
(**A**) 3D block showing regional relief for the top of the mounded drift (D3) and its adjacent moat. (**B**) Thickness map for SU2, and (**C**) surface map for the top of SU2 (D3).

**Figure 4:**
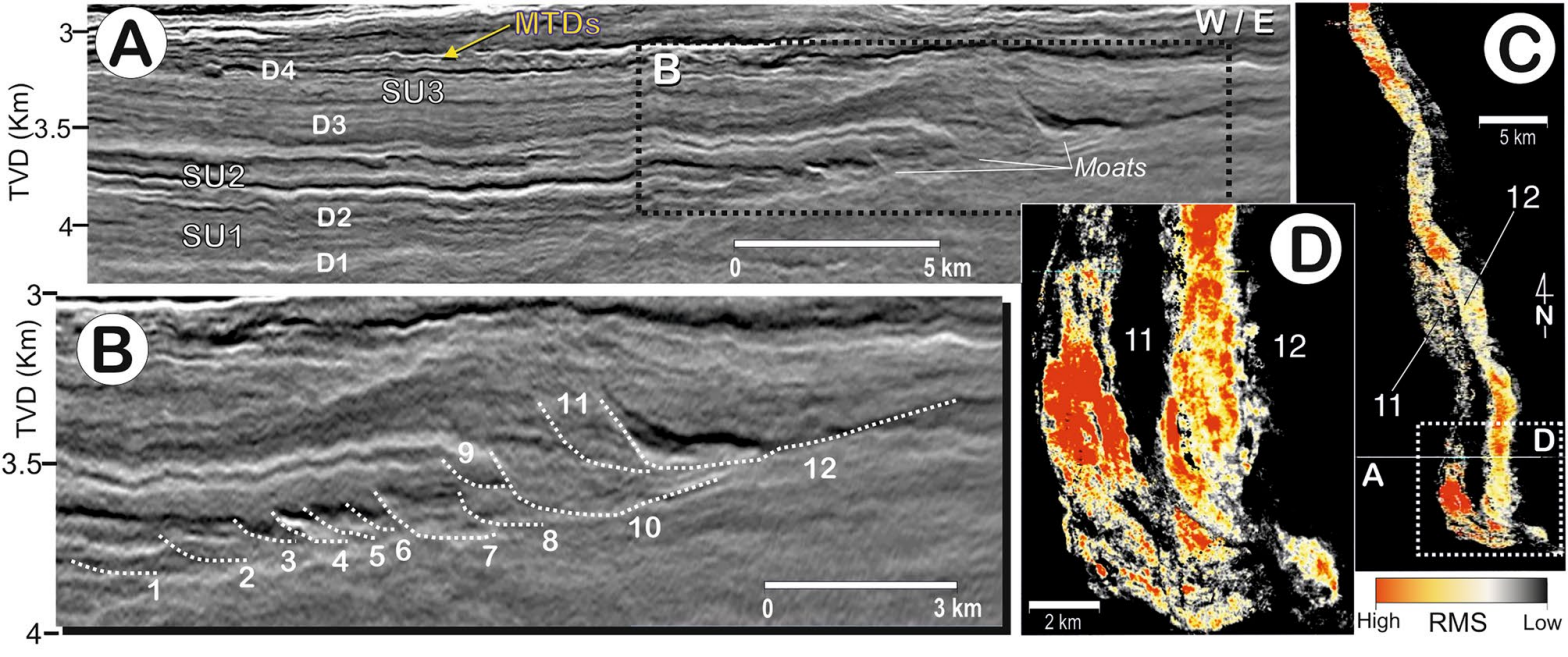
(**A**) 3D *seismic lines* indicating the seismic units, discontinuities and the position of the channels (moats). (**B**) Detail showing the lateral eastward migration of moats. (**C,D**) Root mean square (RMS) amplitude attribute extraction indicating the higher acoustic response of moats 11 and 12, especially where they bend.

Discontinuity D2 is a highly reflective and erosional surface surface that shows evidence of erosion, it separates deposits with a progradational stacking pattern from aggrading ones. SU2 is up to 0.2–0.25 km thick and represents a mounded deposit with an adjacent channel trending N-NW, developing parallel to the slope (Figs. 2 and 3). The internal reflection configuration shows a remarkable sigmoidal to oblique upslope progradation, with a succession of eastward-migrating channel-like features (Figs. 3 and 4). Minor internal discontinuities identified within SU2 consist of higher amplitude reflections (HARs) than those of SU1; as they are related to the channel incisions, laterally the HARs become increasingly expressive towards the channels (Fig. 4). The channel-like features span 2–3 km in width and are up to 40 km in length. They are asymmetric, showing a steeper west flank and shallower east flank, and become wider and with deeper incisions in SU2. The last channelised feature—referred to as channel 12, or the *Anaconda Channel—*is the largest channel in SU2 (Fig. 4).

SU3 represents a regional aggradational and seismically homogeneous tabular unit with an average sedimentary thickness of about 100 m. It is characterised by weak, low-amplitude reflections draped over the underlying drift (Fig. 2). This unit is overlain by packages with high amplitude and chaotic reflection deposits (Fig. 2).

## Chronology, depositional system, paleocurrents and evolutionary stages

The deposits outlined between SU1 to SU3 occur at the foot of the *paleoslope*. The basal erosional surface that defines discontinuity D1 marks the Cretaceous-Paleocene boundary^19^. The SU1 and SU2 correspond to sequence 4 of Delaunay (2018), deposited during the lower Selandian to upper Thanetian (61.6 to 56 Ma)^19^. The D1 represents a prominent unconformity, marking a change in the depositional style along the margin. The incised channel-like features trending SW start at the base of the slope, and are therefore neither continuous upslope (as canyons, gullies, etc.) nor part of any gravitational erosional system. These incisions show similarities to furrows, which commonly trend oblique to the slope and are much narrower and less incised than contourite channels. The furrows are erosive, elongated, sub-parallel features. Overall they are quite regularly spaced, typically a few km in length, a few tens of m wide, and a few tens of cm deep; but in exceptional cases they reach lengths of a few tens of km, thus representing large-scale erosional features^2,6,20^. Sometimes they penetrate coarse gravel and sand substrates^21^ or fine-grained cohesive sediments^22^. Their origin has been associated with small, detached filaments of flow separated from the main core of the bottom current, possibly due to the effects of topography ^6,20^.

Therefore, the observed SW trending channel-like features associated with D1 could be interpreted as furrows running oblique to the paleoslope (Fig. 2). This deviation may record friction between the sea-floor and a water mass, creating Ekman effects in the boundary layer^23^. The bottom current velocity for generating furrows is > 0.3 m s^–1^ in a muddy substrate, 0.6–1.5 m s^–1^ for a sandy one, and > 0.75 m s^–1^ for sandy to gravelly substrate^24^. The association of these furrows with discontinuity D1 indicates bottom current velocities of at least > 0.5 m s^–1^, thus exceeding the bottom current velocity required for the drift that formed later (< 0.25 m s^–1^). Interestingly, furrows often appear in association with the basal^25^ or internal^26^ discontinuities of drifts. Together, these findings offer evidence of higher bottom current velocities during the formation of discontinuities than during drift deposition. Along the drifts the current velocity is weaker, being dominated by fine-grained deposits and muddy contourites, yet mainly by hemipelagic deposits ^27,28^. This variability evidences a switch from a current-dominated setting to anon-current-dominated, or vice versa.

The SU1 is interpreted as a lower Paleocene sheeted drift, based on its internal reflection configuration and shape as determined by Fauguères et al. ^5^ and Brackenridge et al. ^29^. The SU2, which formed during the middle to upper Paleocene, represents a large elongated along-slope mounded morphology having an adjacent continental slope, bounded by an along-slope channel-like feature. This deposit shows characteristics similar to those defined in the literature for elongated, mounded and separated drifts; and the geometry of the adjacent channels and their seismic facies suggest they represent moats, in view of the identification criteria of contouritic features from Fauguères et al. ^5^ and Rebesco et al. ^2^. Because the Coriolis force causes the water mass to veer to the left regionally (southern hemisphere), the flow tends to erode the lower slope and left flank of the moat (down-current) and to construct an elongate separated mounded drift on the right side where the current velocity slackens. The lateral upslope migration of the drift-moat system and some erosion of the adjacent lower slope determine the sedimentary stacking pattern of the mounded and separated drifts (Figs. 1, 2 and 3). Hence, a combination of these large depositional and erosional features would define a CDS that formed during the Paleocene. The relative leftward (westward) position of the drift with respect to the adjacent channel, and thus the slope, provides evidence supporting the influence of the Coriolis force on southwesterly flowing bottom current circulation in the southern hemisphere ^2,5^.

The low amplitude and transparent seismic features of SU3 point to deposition dominated by hemipelagic/pelagic settling (marls). Because D3 corresponds to the Paleocene-Eocene boundary, SU3 is Eocene in age^19^. The upper surface marks the Oligocene boundary, where high amplitude and chaotic reflection deposits—interpreted as mass transport deposits (MTDs)—become dominant (Fig. 2).

The three main seismic units (SU1-SU3) of the Paleogene deposits highlight three evolutionary stages within the CDS: (a) the onset stage (SU1), (b) a drift growth stage (SU2), and (c) a burial stage (SU3). These three stages and their associated large-scale sedimentary architectures are common to drifts of various ages identified along other margins^9,28–35^. Such a coincidence of features and architectures underlines the mechanistic evolution of drifts in response to the long-term behaviour of the water masses responsible for their formation.

Further Paleocene records contain evidence of active deep-water circulation in the South Atlantic^9,18,36^, which persisted as a dominant process until Miocene or even Quaternary times. Nevertheless, obvious contourite features disappeared from the Morondava Basin by the Eocene, and the southwest bottom current indicates an opposite bottom current direction at the base of the slope during the Paleocene as compared with the present-day northeast circulation of deeper water masses (Fig. 1A) that should be further investigated in future research. Cessation of movement along the DFZ and separation of Madagascar from the India-Seychelles block^37^ likely contributed to the new depositional system in which bottom currents shaped the deep-water margin during the Paleocene. The decay of bottom currents at the Paleocene-Eocene boundary and the initiation of MTDs at the beginning of the Oligocene coincided with global climatic and plate tectonic changes that included plate boundary rearrangement and co-evolving deep-ocean circulation^38–40^.

## The contourite channel-like features

Owing to their smaller size at the base of SU2 relative to the last channel along the upper surface (the *Anaconda Channel*) and their eastward migration, the contourite channel-like features identified are categorized as “moats”. Moats are channels parallel to the slope, originated by non-deposition and localised erosion beneath the core of the bottom current. Moats differ from other contourite channels (or turbiditic channels) due to their genetic relationship with one type of drift, the elongated, mounded, and separated contourite drift, representing prolonged, high-energy current activity^2,5^.

The moat stacking pattern is very intriguing. During the deposition of SU1 the moats are small (~ 2 km wide, with 100 m of incision) yet progressively become larger (~ 2.5 to 3 km wide and 0.15 km of incision) at the base of SU2, then even larger in the upper part of SU2 (~ 5 km wide and > 0.2 km of incision). Coevally, moats migrate laterally during SU1, despite some aggradational components and thicker sedimentary infilling during SU2. The change in moat dimensions can be correlated to the adjacent drift morphology (Figs. 2, 3 and 4), showing changes from a sheeted, to a slightly mounded, and then to a highly pronounced mounded shape. The long-term evolution of moats over time (especially during the growth stage) coincided with an expansion and intensification of deep-water circulation that eventually modulated the formation of the drifts and the backstepping stacking pattern of the channels. The moat architecture provides evidence of remarkable incision over shorter time periods, reflecting accelerating (incision) and decelerating (infilling) or absent bottom currents (Fig. 4). The record furthermore shows the influence of an intermittent bottom current during the deposition of SU2 that increases over time, in tandem with the construction of the mounded drift. Similar upslope progradation of moats occurs in other CDSs^5^. The previously identified moats indicate average bottom current velocities between 0.6 and 1 m s^–1^^41^. Recent studies^42,43^ likewise document the intermittent activity of bottom currents during the onset and evolution of contourite channels.

Vertical profiles from the 3D seismic data reveal high-amplitude reflections (HARs) with Root Mean Square (RMS) extractions clearly evidencing high-amplitude anomalies within these moats, particularly where they bend (Fig. 4). Such anomalies might indicate coarser sediment along the moats, as evoked for other contourite channels and moats within CDS known to contain vast quantities of well-sorted sands^21,26,29,41,42^. Present day contourite channels in the Gulf of Cadiz^21^ and late Miocene channels exposed in Morocco^42,43^ confirm that extensive sandy deposits typify these features. In all these examples, the sandier deposits are brought into the moats by gravitational processes; and once inside the moat they are reworked, being laterally transported and deposited by a higher velocity core of the current along it^41–43^. The petrophysical characteristics of such deposits coincide with those of reservoirs, making them potential targets for hydrocarbon exploration or underground CO_2_ and energy storages. Mounded drifts have high proportions of mud, while deposits forming during the burial stage (SU3) represent sediment drapes, which should also have high mud-to-sand ratios. Altogether, these deposits possess the characteristics needed to form a good seal. In summary, then, the moats identified in the Paleogene sedimentary succession of the Morondava Basin contain HARs—potentially representing a sandier deposit which could be reservoir target in a petroleum play, given their position relative to Triassic, Jurassic and Cretaceous source rocks ^17^. Still, this hypothesis can only be confirmed by drilling and subsequent study.

## Conclusions

A Contourite Depositional System (CDS) has been identified in Paleocene sediments of the Morondova Basin. This CDS documents a long-term, progressive increase in bottom current activity along the foot of the slope until the Eocene. Bottom currents and the CDS developed coevally with global plate tectonic, climatic and oceanographic events. The final CDS morphology is determined by the basal and upper boundaries, as well as internal discontinuities that document higher current velocities shaping the drift and adjacent erosive channels (moats). These discontinuities and the architecture of erosional features provide evidence of the shorter-term, intermittent behaviour of currents during CDS formation. Such intermittent events also determined the distribution of sedimentary facies, in particular the sandier deposits along the moats that hold potential as reservoirs for CO_2_ storage and hydrocarbon exploration. Future research using 3D seismic datasets to evaluate CDSs at a higher spatial and temporal resolution will help further elucidate depositional signs of water mass dynamics.

## Methods

The dataset, which extends over the Grand Prix block, was provided by the OMV company and acquired by WesternGeco in 2015. Acquisition occurred onboard the WG Magellan vessel, with a nominal fold of 80, a record length of 8192 ms, a sample interval of 2 ms, and recording filters of 2 Hz, 18 dB/oct (low-cut) and 200 Hz, 477 dB/oct (high-cut). The seismic source was appraised by Tuned Bolt Airgun Array and received by 12, 8 km long streamers with a separation of 100 m. Data processing was executed by La Compagnie Générale de Géophysique (CGG) in 2016 with a post-time migrated stack in time and depth cube (50 m trace spacing). Age assignments within the deep-water basin fill were established by correlating the stratigraphic tops from adjacent wells drilled on the shelf (Fig. 1): Ankilimanarivo-2; Chesterfield-1; Cap Saint Andre-1 and -2; Eponge-1; Heloise-1; Morombe-1; Morondava-1; and Vaucluse-1^17^.

## Data Availability

The data that support the findings of this study are available from OMV and OMNIS, but restrictions apply to their availability, used under license for the current study, and therefore not publicly available. Some data are, however, available from the authors upon reasonable request and with permission of OMV and OMNIS.
